# The Prevalence and Possible Association of Different Types of Temporomandibular Disorders Among Young Adult Patients With Anterior and/or Posterior Crossbite: A Cross-Sectional Study

**DOI:** 10.7759/cureus.74047

**Published:** 2024-11-19

**Authors:** Hanan Ahmad Rame Kamar Alden, Kinda Sultan, Mohammad Y. Hajeer

**Affiliations:** 1 Department of Orthodontics, Faculty of Dentistry, University of Damascus, Damascus, SYR

**Keywords:** anterior crossbite, clinical examination, disc displacement, muscle disorders, odds ratio, posterior crossbite, temporomandibular disorders, temporomandibular joint (tmj) disorders, tmj

## Abstract

Objectives: This study aimed to investigate the prevalence and the possible association of the different types of temporomandibular disorders among young adult patients with anterior and/or posterior crossbites.

Methods: This cross-sectional study included 584 individuals (259 male participants and 325 female participants) aged 18-29. The temporomandibular joint examination was conducted according to the Research Diagnostic Criteria for Temporomandibular Disorders (RDC/TMD) Axis I tests. In addition, dental and occlusal examinations were conducted to investigate the presence or absence of anterior and/or posterior crossbites. A chi-square test was used to evaluate any possible association between TMDs and gender and crossbite groups. A binary logistic regression analysis was performed to compute the odds ratios and assess the predictability of TMDs based on the presence or absence of crossbites.

Results: Here, 47.1% of participants had a positive TMD. The presence of muscle disorder, disc displacement, and other joint disorders among participants was 16.4%, 26.7%, and 25.7%, respectively. The prevalence of TMDs in the female participants was significantly greater than in male participants (P= 0.046). There was a significant difference in the prevalence of disc displacements among crossbite groups (P= 0.026), and the anterior crossbite group had the highest prevalence (41%). There was no significant difference in the prevalence of muscle disorder and other joint disorders among crossbite groups (P= 0.149; P= 0.052, respectively). According to the binary logistic regression, only an anterior crossbite was significantly associated with higher predictability for disc displacement and other joint disorders (OR= 2.4; P= 0.008, OR= 2.34; P= 0.01, respectively). The presence of only a posterior crossbite was significantly associated with higher predictability for the occurrence of the disc displacement (OR= 1.58; P= 0.03).

Conclusion: The risk of development of disc displacement may be increased by the presence of an anterior or posterior crossbite. Also, the risk of developing other joint disorders may be increased by the presence of only an anterior crossbite.

## Introduction

Temporomandibular disorders (TMDs) are a collective term containing a group of different problems affecting the masticatory muscles, the temporomandibular joint (TMJ), and their associated structures [1], whether these problems are in structure or in function [2]. TMDs are considered the first cause of non-dental pain in the orofacial region [3] and the second most common disease of the musculoskeletal system (after chronic lower backache) [4]. Furthermore, TMDs affect all age groups, but their prevalence is more widespread among adults than children and adolescents; moreover, symptoms are more frequent between the ages of 17-30 years [5].

The etiology of TMDs is complex and influenced by several factors such as hereditary factors, hormonal factors, systemic disease, trauma [6], head posture [7], oral behaviors [8], race [9], psychological factors [10], and malocclusion. Furthermore, malocclusion may play more than one role in the development of TMDs, as it may be a predisposing, prolonging, or even initiating factor [6]. However, despite these different predictability roles that malocclusion may play, there is still great controversy about the relationship between TMDs and some types of malocclusion, especially anterior and posterior crossbites.

Many studies have confirmed a relationship between TMDs and the types of crossbites. Chen and his colleagues found that the presence of an anterior crossbite affects the morphology of the TMJ [11]. Also, Amer et al. discussed the role of the posterior crossbite in altering the function of the component structures of the TMJ [12]. Moreover, several studies recommended early treatment for these patterns of malocclusion because they constituted risk factors for the development of TMDs [13,14]. On the other hand, other studies have supposed that crossbites have no role in the occurrence of TMDs [15]. So, there is a need for more investigations into the relationship between TMDs and the different types of crossbites. Therefore, this cross-sectional study was conducted to investigate the prevalence of various types of TMDs among young adult patients with anterior crossbites, posterior crossbites, or a combination of both and to investigate the possible association between the occurrence of TMDs and the presence of anterior and/or posterior crossbites.

## Materials and methods

Study design and ethical consideration

A cross-sectional study was undertaken between February 2023 and March 2024 at the Faculty of Dentistry, University of Damascus, Syria. Before participating in the study, all participants were required to give written informed consent. This research project was funded by the University of Damascus, Syria (Reference no: 501100020595), and ethical approval was obtained from the Local Research Ethics Committee of the Faculty of Dentistry, University of Damascus (UDDS-11723022023/SRC-299).

Study sample calculation

The minimum required sample size was determined based on a previous related study [16]. The “Epitools” website [17] calculated the sample size. The minimum sample size required was 556 individuals, with a 95% confidence level and a 5% margin of error.

Patient recruitment and the inclusion/exclusion criteria

The inclusion criteria included the following items: 18-29-year-old patients, the presence of all permanent teeth (except the third molars), absence of any general joint diseases, absence of a previous diagnosis of a pathological state in the ear, and absence of craniofacial deformities or congenital/developmental disorders.

The exclusion criteria included the presence of current or previous orthodontic treatment, previous or current treatment for TMJ disorders, medication with muscle relaxants in the current period, a history of previous exposure to a severe traumatic injury to the face or jaw area, and cases of dislocation or subluxation of the TMJ.

Sample collection

After evaluating 896 individuals who attended the Faculty of Dentistry at the University of Damascus for general routine dental examination and were aged 18-29, 672 met the inclusion criteria. However, 584 individuals agreed to participate after receiving a sheet with information about the study's objectives and the examination procedures. Written and oral consent was obtained from each individual participating in this study. All examination procedures were completed with the participant seated in a dental chair before the principal researcher (HQ-A).

Temporomandibular joint clinical examination and diagnosis

**Figure 1:**
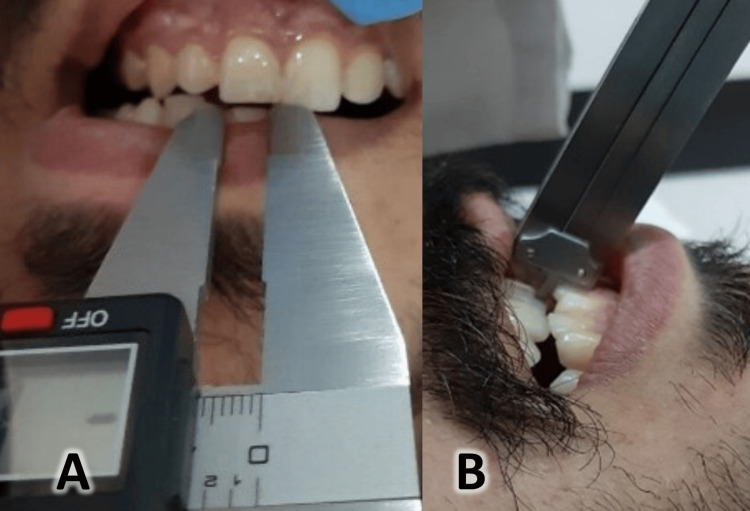
Measuring mandibular excursive movements: A: Measuring mandibular lateral excursion and B: measuring mandibular protrusion.

**Figure 2:**
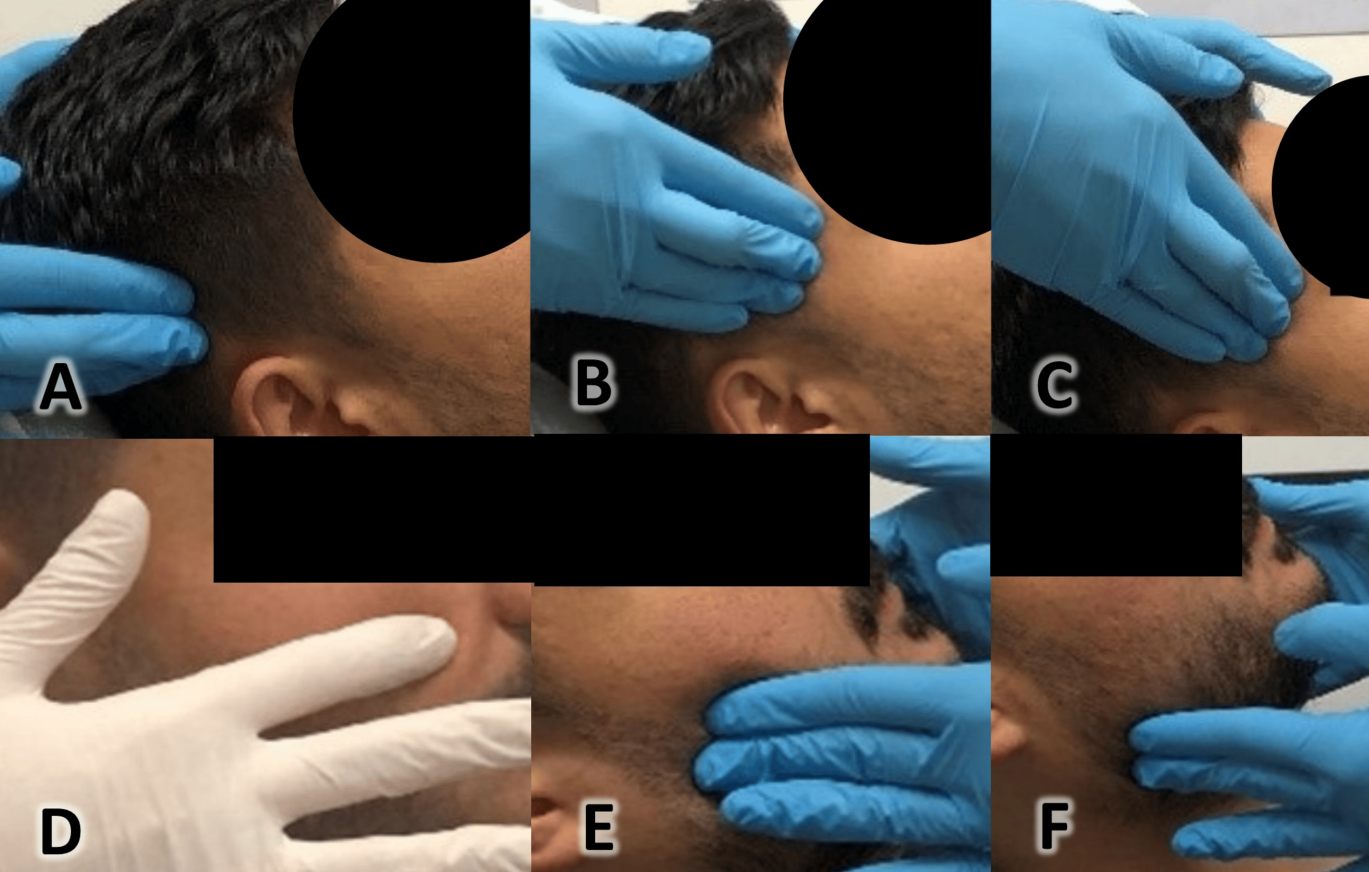
Extraoral muscle palpation. A: Palpation of the posterior temporalis muscle, B: Palpation of the middle temporalis muscle, C: Palpation of the anterior temporalis muscle, D: Palpation of the origin of the masseter muscle, E: Palpation of the body of the masseter muscle, and F: Palpation of the insertions of the masseter muscle.

The TMJ examination was conducted according to the Research Diagnostic Criteria for Temporomandibular Disorders (RDC/TMD) Axis I tests [18,19], which consists of 10 items including three subjective questions, defining the opening pattern, testing of mandibular movements (the vertical range of motion of mandibular, lateral excursion, and protrusion; Figure 1), investigating about joint sound and pain at any mandibular movements, and the associated muscles and joint examination for tenderness by palpation (the palpation was done with one pound of pressure for the intraoral muscles and joints, and two pounds of pressure for the extraoral muscles for two to five seconds; Figure 2).

The temporomandibular joint disorders diagnostic algorithm, which was recommended by Dworkin and LeResche and was described clearly among International Network for Orofacial Pain and Related Disorders Methodology INFORM [18,19], was applied to the outcomes of Axis I of RDC/TMD to determine the presence/absence of the muscle disorders (including myofascial pain, myofascial pain with limited opening), the disc displacements (including disc displacement with reduction, disc displacement without reduction with limited opening, and disc displacement without reduction without limited opening), and the other joint disorders (including arthralgia, osteoarthritis, and osteoarthrosis). Additionally, the participant was considered positive-TMD if there was a presence of one or more types of TMDs and considered negative-TMD if there was the absence of all types of TMDs.

Dental and occlusal examination and diagnosis

An anterior crossbite is defined when the position of one or more of the maxillary anterior teeth is palatal relative to the mandibular anterior teeth [20]. A posterior crossbite was defined when at least one buccal cusp of maxillary posterior teeth has been in contact with the central fossae of mandibular posterior crossbite teeth at the intercuspal position [21]. Finally, the participants were classified into four groups: Group 1: Posterior Crossbite (including participants with only posterior crossbite). Group 2: Anterior Crossbite (including participants with only anterior crossbite). Group 3: Mixed Crossbite (including participants with both anterior and posterior crossbite). Group 4: No Crossbite (including participants who have no crossbite)

Outcome measures

The following types of data were collected: (1) patient's gender, (2) age, (3) presence or absence of muscle disorders, (4) presence or absence of disc displacements, (5) presence or absence of other joint disorders, (5) presence or absence of TMDs, and (6) the type of crossbite (posterior crossbite, anterior crossbite, a combination of both (mixed crossbites), or absence of a crossbite).

Statistical analysis

All statistical analysis was performed using IBM SPSS Statistics for Windows, Version 23 (Released 2015; IBM Corp., Armonk, New York, United States). Descriptive statistics were achieved for all studied variables, including percentages and frequencies for categorical variables and means for continuous ones. The chi-square test was used appropriately for categorical variables. A binary logistic regression analysis was used to compute the odds ratio to identify predictors of occurrence of TMDs and subtypes of TMDs.

## Results

Five hundred eighty-four individuals who agreed to participate in the current study were enrolled; 55.7% (325) were female participants. The mean age of the study participants was 22.3 years, with a standard division of 3.06 years; 47.1% of participants had a positive TMD. The presence of muscle disorders, disc displacement, and other joint disorders (including arthralgia, osteoarthritis, and osteoarthrosis) among participants was 16.4%, 26.7%, and 25.7%, respectively. Almost two-thirds (58.6%) of the study participants did not have any crossbite (No Crossbite group), 7.5% had only anterior crossbite (Anterior Crossbite group), 24.5% had only posterior crossbite (Posterior Crossbite group), and 9.2% had both anterior and posterior crossbites (Mixed Crossbite group) (Table 1).

**Table 1:** Baseline sample characteristics SD: standard division, TMD: temporomandibular disorder, XB: crossbite * in years.

Variable	Categories	N (%)
Sex	Male	299 (44.3)
	Female	342 (55.7)
Age (Mean+SD)*		22.49±3.06
TMD	Positive	275 (47.1)
	Negative	309 (52.9)
Muscle disorder	Presence	96 (16.4)
	Absence	488 (83.6)
Disc displacement	Presence	156 (26.7)
	Absence	428 (73.3)
Other joint disorders	Presence	190 (25.7)
	Absence	434 (74.3)
Crossbite	Posterior XB	144 (24.5)
	Anterior XB	44 (7.5)
	Mixed XB	54 (9.2)
	No XB	312 (58.6)

The prevalence of TMDs in the female participants was significantly greater than in male participants (50.7% and 42.2%, respectively) (P-value=0.046). Additionally, there was a significant difference in the prevalence of muscle disorder among genders (P-value=0.002), but there was no significant difference in the prevalence of disc displacements and other joint disorders among genders (P-value=0.549; P-value=0.214, respectively). Moreover, there was no significant difference in the prevalence of crossbite among genders (P-value=0.063; Table 2).

**Table 2:** Descriptive statistics of the prevalence and the association of temporomandibular disorders, muscle disorders, disc displacements, other joint disorders, and crossbites with respect to gender (Female: 325 patients and Male: 259 patients). a: Chi-square test * Significance at P-value ≤ 0.05; N: number of cases. TMD: temporomandibular disorder, XB: crossbite

Deformity or Disorder	Status	Gender		Pearson's Chi-square	P-value^ a^
		Female; N (%)	Male; N (%)		
TMD	Positive	165 (50.7%)	110 (42.2%)	3.984	0.046^*^
	Negative	160 (49.3%)	149 (57.8%)		
Muscle disorder	Presence	67 (20.6%)	29 (11.1%)	9.308	0.002^*^
	Absence	258 (79.4%)	230 (88.9%)		
Disc displacement	Presence	90 (27.6%)	66 (25.4%)	0.359	0.549
	Absence	235 (72,3%)	193 (76.6%)		
Other joint disorders	Presence	90 (27.6%)	60 (23.1%)	1.547	0.214
	Absence	235 (72,3%)	199 (76.9%)		
Crossbite	Posterior XB	84 (25.8%)	60 (23.6%)	6.906	0.075
	Anterior XB	18 (5.5%)	26 (10%)		
	Mixed XB	25 (7.6%)	29 (11.1%)		
	No XB	198 (60.9%)	144 (55.5%)		

There was a significant difference in the prevalence of disc displacements among crossbite groups (P-value=0.026). The prevalence of disc displacements among participants without crossbites was 22.8%, while it was 41% in those with anterior crossbite, it was 31.9% in those with posterior crossbite, and it was 25.9% in those with both anterior crossbite and posterior crossbite, but there was no significant difference in the prevalence of muscle disorder and other joint disorders among crossbite groups (P-value=0.149; P-value=0.052, respectively; Table 3).

**Table 3:** Descriptive statistics of the prevalence and the association of TMDs, muscle disorders, disc displacements, and other joint disorders with respect to the type of crossbite. a: Chi-square test TMD: temporomandibular disorder, XB: crossbite * Significance at P-value ≤ 0.05 † Posterior XB: 144 participants, Anterior XB: 44 participants, Mixed XB: 54 participants, No XB: 342 participants.

Disorder	Status	Crossbite†				Pearson's Chi-square	P-value^a^
		Posterior XB N (%)	Anterior XB N (%)	Mixed XB N (%)	No XB N (%)		
TMD	Positive	69 (47.9%)	25 (56.8%)	29 (53.7%)	152 (44.4%)	3.620	0.306
	Negative	75 (52%)	19 (43.1%)	25 (46.3%)	190 (55.5%)		
Muscle disorder	Presence	27 (18.7%)	11 (25%)	11 (20.3%)	47 (13.7%)	5.325	0.149
	Absence	117 (81.2%)	33 (75%)	43 (79.6%)	295 (86.2%)		
Disc displacement	Presence	46 (31.9%)	18 (41%)	14 (25.9%)	78 (22.8%)	9.225	0.026^*^
	Absence	98 (68%)	26 (59%)	40 (74%)	264 (77.1%)		
Other joint disorders	Presence	35 (24.3%)	19 (43.1%)	14 (25.9%)	82 (23.9%)	7.725	0.052
	Absence	109 (75.7%)	25 (56.8%)	40 (74%)	260 (76%)		

According to the binary logistic regression, the presence of only an anterior crossbite was significantly associated with a higher predictability for the occurrence of disc displacement and other joint disorders (OR 2.4; P-value=0.008, OR 2.34; P-value=0.01, respectively), but the association between the presence of only an anterior crossbite and a higher predictability for the occurrence of the muscle disorder was borderline significant (OR 2.09; P-value=0.053). Moreover, the presence of only a posterior crossbite was significantly associated with a higher predictability for the occurrence of the disc displacement (OR 1.58, P-value=0.03), and the presence of only a posterior crossbite was associated with a higher predictability for the occurrence of the muscle disorder and other joint disorders, but this association was not significant (OR 1.48; P-value=0.16, OR 1.01; P-value=093, respectively). Similarly, the presence of mixed crossbite was associated with higher predictability for the occurrence of muscle disorder, disc displacement, and other joint disorders, but this association was not significant (OR 1.60; P-value=0.2, OR 1.18; P-value=0.61, OR 1.11; P-value=0.79, respectively; Table 4).

**Table 4:** Binary logistic regression for prediction of the occurrence of the TMDs, muscle disorders, disc displacements, and other joint disorders in patients with posterior crossbites, anterior crossbites, or mixed crossbites. † The class reference in the logistic regression was "No XB", CI: confidence interval, Coef.: coefficient OR: Odds ratio, TMD: temporomandibular disorder, XB: crossbite * Significance at P-value ≤ 0.05 using binary logistic regression.

	TMD				Muscle disorder				Disc displacement				Other joint disorders			
Type of Crossbite^†^	B Coef.	OR	95% CI of OR	P-value	B Coef.	OR	95% CI of OR	P-value	B Coef.	OR	95% CI of OR	P-value	B Coef.	OR	95% CI of OR	P-value
Posterior XB	0.140	1.150	0.778-1.699	0.483	0.370	1.448	0.862-2.435	0.161	0.463	1.589	1.032-2.447	0.036	0.018	1.018	0.646-1.604	0.938
Anterior XB	0.498	1.645	0.873-3.099	0.124	0.738	2.092	0.990-4.423	0.053	0.852	2.343	1.221-4.497	*0.010	0.880	2.410	1.263-4.598	*0.008
Mixed XB	0.372	1.450	0.815-2.579	0.206	0.474	1.606	0.774-3.333	0.262	0.169	1.185	0.613-2.290	0.614	0.104	1.110	0.575-2.141	0.756

## Discussion

The etiology of TMDs is multifactorial, and their symptoms vary and may become chronic, which affects the quality of patient life [6]. The diversity of symptoms and disorders has led to diverse treatment options, which sometimes fail to reach the desired results, like the therapy with occlusal splints, which needs a high degree of precision in the selection between their various designs, as the splint design affects in the masticatory muscle activation [22]. Also, occlusal equilibration therapy has to be done with minimal invasive occlusal remodeling and high accuracy to obtain balanced occlusion and reduce the intensity of facial pain associated with chronic TMDs [23]. So, there is a need to identify the possible etiological factors and determine their role in developing TMDs to establish the best treatment. By reviewing the literature, it has become clear that the relationship between crossbite and TMJ disorders is still confusing and ambiguous, and the role of each type of crossbite in the development of TMDs has not been precisely and consistently defined. Therefore, this study investigated the possible relationship between crossbite patterns and TMDs.

In this study, 47.1% of participants have one or more types of TMDs; this was approximately consistent with many previous studies [16,24], and a systematic review conducted by Alrizqi and Aleissa revealed that the prevalence of TMDs among adults ranges from 30 to 50% [25], but 34% was the rate of prevalence among Syrian adults which was lower than the result of this study [26]. This discrepancy may be attributable to the use of solely questionnaire-based assessment tools without any clinical examination.

This study included 18-29-year-old patients because the prevalence of TMDs is highest among patients aged 20-40 [27], and according to Egermark et al. [5] in their 20-year longitudinal study, the symptoms are more frequent between the ages of 17-30. Moreover, the protocol of the current study included TMJ clinical examination with no X-ray or other assistant diagnostic tools, so there was a need to identify the signs and symptoms more clearly to identify among this age group [5].

This study observed that the prevalence of TMDs was significantly greater among female participants than male ones. Gender differences in hormones, physical structure, brain chemistry, and metabolism affect the biological mechanism for receiving, transmitting, and modulating pain [28]. This result agrees with many previous studies that assessed the prevalence of TMDs among different genders [25,28-31].

The present study revealed that individuals without crossbites exhibited the lowest prevalence of TMDs compared to those with posterior and/or anterior crossbites. Additionally, it found higher predictability for muscle disorders, disc displacements, and other joint disorders among individuals with different types of crossbite. Furthermore, an anterior or posterior crossbite was significantly associated with higher predictability for disc displacement, and only the anterior crossbite was significantly associated with higher predictability for other joint disorders. Also, the individuals with anterior crossbites had higher predictability for the occurrence of subtypes of TMDs compared to those with posterior or mixed crossbites. These findings may be due to individuals with anterior crossbite having a deficiency of the anterior inclination of the condyle and no incisal guidance. Moreover, the incisal guidance relates to the TMJ development, stabilizing the condyle. So, the absence of incisal guidance has huge effects on the condyle movement and mandibular movement and alters the neuromuscular reflex, which causes abnormal movements of the lower jaw, which may put pressure on the temporomandibular joint and affect the position and shape of TMJ component structures, which causes structure degeneration and slows down TMJ remodeling, which leads to TMDs [11,31].

Furthermore, individuals with a posterior crossbite have an altered occlusal/morphological relationship between the lower and upper dentition, as when the lower jaw moves to reach the intercuspal position, it causes an asymmetry in the masseter muscles and variation in the relationship of the condyle with the articular fossa which maybe causes a disc displacement [12]. Moreover, these results are consistent with many previous studies [16,32-35]. Barrera-Mora et al. suggested that the anterior crossbite is considered a risk factor for development of disc displacement and joint pain [14]. Also, the early urgent treatment for posterior crossbite was recommended by Thilander et al. [13]. Moreover, Mélou et al. found a significant association between TMDs and the interferences in lateral excursions [36]. Furthermore, according to Pullinger et al. [37], who applied a multiple logistic regression analysis to investigate the relative odds of occurrence of TMD with the presence of many occlusal features, the possibility of occurrence of disc displacement in individuals with posterior crossbite ranges from 3.3 to 1 comparison to the ones without posterior crossbite, which is accordance with the results of the current study.

On the other hand, according to Gesch et al. [32], who also applied multiple logistic regression analysis, there were weak associations between anterior or posterior crossbite bite and the signs of TMDs, but this study included ages up to 80 years, and the increasing number of missing teeth with age (especially in subjects over 50) may cause differential effects on the TMJ. Also, the findings of the current study did not consent with the conclusions from the study by Al-Khatieeb et al. [38] and study by Aboalnaga et al. [15], which found no correlation between posterior crossbites and types or parameters of TMDs; this variation from our results may be due to the variance of the study design, and absence of the control group in the study by Aboalnaga et al. Also, Myllymaik et al. [39] concluded there was no significant association between anterior crossbites and the related sound of disc displacement, but this study was longitudinal, and some of the participants had an orthodontic treatment.

Limitations of the current work

The current study used clinical examinations and questionnaires to diagnose TMDs without an MRI or CT. MRI and CT may help to confirm the diagnosis of TMDs, especially the degenerative disorder, and to determine the type of posterior crossbite (skeletal or dental). Furthermore, this study was based on patients attending the Faculty of Dentistry. The current study was not conducted on an ordinary population living in normal circumstances, who may have had different characteristics from those attending teaching hospitals and medical centers for dental treatment.

## Conclusions

The prevalence of TMDs is greater among female adults compared to male adults, specifically for muscle disorders. Furthermore, the prevalence of disc displacements is significantly different among adults with or without crossbites. Moreover, the presence of anterior or posterior crossbites may increase the risk of developing disc displacements. Also, the risk of developing other joint disorders may be increased by the presence of anterior crossbites but not by the presence of posterior crossbites. Crossbites did not significantly influence the risk of developing muscle disorders. The findings of this study highlighted the urgent need for early treatment for the different types of crossbites as they considered predictable risk factors for the development of TMDs. 

## References

[1] Durham J (2008). Temporomandibular disorders (TMD): an overview. Oral Surg.

[2] Ohrbach R, Sharma S (2024). Temporomandibular disorders: definition and etiology. Semin Orthod.

[3] Bagis B, Ayaz EA, Turgut S, Durkan R, Özcan M (2012). Gender difference in prevalence of signs and symptoms of temporomandibular joint disorders: a retrospective study on 243 consecutive patients. Int J Med Sci.

[4] Ferrando M, Andreu Y, Galdón MJ, Durá E, Poveda R, Bagán JV (2004). Psychological variables and temporomandibular disorders: distress, coping, and personality. Oral Surg Oral Med Oral Pathol Oral Radiol Endod.

[5] Egermark I, Carlsson GE, Magnusson T (2001). A 20-year longitudinal study of subjective symptoms of temporomandibular disorders from childhood to adulthood. Acta Odontol Scand.

[6] Chisnoiu AM, Picos AM, Popa S, Chisnoiu PD, Lascu L, Picos A, Chisnoiu R (2015). Factors involved in the etiology of temporomandibular disorders - a literature review. Clujul Med.

[7] Visscher CM, De Boer W, Lobbezoo F, Habets LL, Naeije M (2002). Is there a relationship between head posture and craniomandibular pain?. J Oral Rehabil.

[8] Tian Y, Tan Y, Yang M (2024). The association between specific oral behaviors and the number of temporomandibular disorder symptoms in the general population: a cross-sectional study. J Pain Res.

[9] Magalhães BG, de-Sousa ST, de Mello VV, da-Silva-Barbosa AC, de-Assis-Morais MP, Barbosa-Vasconcelos MM, Caldas-Júnior AD (2014). Risk factors for temporomandibular disorder: binary logistic regression analysis. Med Oral Patol Oral Cir Bucal.

[10] Felin GC, Tagliari CV, Agostini BA, Collares K (2024). Prevalence of psychological disorders in patients with temporomandibular disorders: a systematic review and meta-analysis. J Prosthet Dent.

[11] Chen Y, Wang J, Li Y (2022). Age-related variations in position and morphology of the temporomandibular joint in individuals with anterior openbite and crossbite: a multi-cross-sectional comparative study. BMC Oral Health.

[12] Amer NM, Aboalnaga AA, Salah Fayed MM, Labib AH (2019). Transverse malocclusion and temporomandibular disorders: verification of the controversy. J Oral Facial Pain Headache.

[13] Thilander B, Rubio G, Pena L, de Mayorga C (2002). Prevalence of temporomandibular dysfunction and its association with malocclusion in children and adolescents: an epidemiologic study related to specified stages of dental development. Angle Orthod.

[14] Barrera-Mora JM, Espinar Escalona E, Abalos Labruzzi C, Llamas Carrera JM, Ballesteros EJ, Solano Reina E, Rocabado M (2012). The relationship between malocclusion, benign joint hypermobility syndrome, condylar position and TMD symptoms. Cranio.

[15] Aboalnaga AA, Amer NM, Elnahas MO (2019). Malocclusion and temporomandibular disorders: verification of the controversy. J Oral Facial Pain Headache.

[16] Khayat N, Winocur E, Kedem R, Winocur Arias O, Zaghal A, Shpack N (2021). The prevalence of temporomandibular disorders and dental attrition levels in patients with posterior crossbite and/or deep bite: a preliminary prospective study. Pain Res Manag.

[17] (2024). Epitools-Epidemiological Calculators. http://epitools.ausvet.com.au.

[18] Dworkin SF, LeResche L (1992). Research diagnostic criteria for temporomandibular disorders: review, criteria, examinations and specifications, critique. J Craniomandib Disord.

[19] (2023). INFORM: International Network for Orofacial Pain and Related Disorders Methodology. https://ubwp.buffalo.edu/rdc-tmdinternational/tmd-assessmentdiagnosis/rdc-tmd/.

[20] Daskalogiannakis J, Miethke RR, McNamara JA (2000). Glossary of Orthodontic Terms. Quintessence, Berlin.

[21] Phulari BS (2011). Orthodontics: Principles and Practice. Orthodontics: principles and practice.

[22] Seiler A, Lukic N, Özcan M, Kazimi M, Kaldas M, Gallo LM, Colombo V (2024). Temporomandibular joint space variation and masticatory muscle activation during clenching with full versus partial covering occlusal splints. Clin Oral Investig.

[23] Santana-Penín U, Santana-Mora U, López-Solache A (2023). Remodeling dental anatomy vs sham therapy for chronic temporomandibular disorders. A placebo-controlled randomized clinical trial. Ann Anat.

[24] Minervini G, Mariani P, Fiorillo L, Cervino G, Cicciù M, Laino L (2022). Prevalence of temporomandibular disorders in people with multiple sclerosis: A systematic review and meta-analysis. Cranio.

[25] Alrizqi AH, Aleissa BM (2023). Prevalence of Temporomandibular Disorders Between 2015-2021: A Literature Review. Cureus.

[26] Issa N, Baherly N (2015). Mayhoube MJIJoBE: Prevalence of Symptoms of Temporomandibular Joint Disorder in Lattakia-Syria. Int J Bio Eng.

[27] Lövgren A, Häggman-Henrikson B, Visscher CM, Lobbezoo F, Marklund S, Wänman A (2016). Temporomandibular pain and jaw dysfunction at different ages covering the lifespan--A population based study. Eur J Pain.

[28] Bueno CH, Pereira DD, Pattussi MP, Grossi PK, Grossi ML (2018). Gender differences in temporomandibular disorders in adult populational studies: A systematic review and meta-analysis. J Oral Rehabil.

[29] Da-Cas CD, Valesan LF, Nascimento LP (2024). Risk factors for temporomandibular disorders: a systematic review of cohort studies. Oral Surg Oral Med Oral Pathol Oral Radiol.

[30] Yap AU, Liu C, Lei J, Park JW, Kim SH, Lee BM, Fu KY (2023). DC/TMD axis I subtyping: generational and gender variations among East Asian TMD patients. BMC Oral Health.

[31] Progiante PS, Pattussi MP, Lawrence HP, Goya S, Grossi PK, Grossi ML (2015). Prevalence of temporomandibular disorders in an adult Brazilian community population using the research diagnostic criteria (Axes I and II) for temporomandibular disorders (The Maringá Study). Int J Prosthodont.

[32] Gesch D, Bernhardt O, Kocher T, John U, Hensel E, Alte D (2004). Association of malocclusion and functional occlusion with signs of temporomandibular disorders in adults: results of the population-based study of health in Pomerania. Angle Orthod.

[33] Karaman A DD, MSc MSc, Buyuk SK (2022). Evaluation of temporomandibular disorder symptoms and oral health-related quality of life in adolescent orthodontic patients with different dental malocclusions. Cranio.

[34] Jain S, Chourse S, Jain D (2018). Prevalence and severity of temporomandibular disorders among the orthodontic patients using Fonseca's questionnaire. Contemp Clin Dent.

[35] Yan Q, Liao L, He D (2024). Risk factors associated with temporomandibular joint disorder: a mendelian randomization analysis. J Oral Rehabil.

[36] Mélou C, Leroux L, Bonnesoeur M, Le Padellec C, Bertaud V, Chauvel-Lebret D (2024). Relationship between natural or iatrogenic malocclusions and temporomandibular disorders: a case control study. Cranio.

[37] Pullinger AG, Seligman DA, Gornbein JA (1993). A multiple logistic regression analysis of the risk and relative odds of temporomandibular disorders as a function of common occlusal features. J Dent Res.

[38] Al-Khatieeb MM, Nissan LM, Al-Labban YR, Abid M (2024). Occlusal features and temporomandibular joint disorder: a cross-sectional study. Int J Dent.

[39] Myllymäki E, Heikinheimo K, Suominen A, Evälahti M, Michelotti A, Svedström-Oristo AL, Rice DP (2023). Longitudinal trends in temporomandibular joint disorder symptoms, the impact of malocclusion and orthodontic treatment: a 20-year prospective study. J Oral Rehabil.

